# Relevance of Neuroinflammation and Encephalitis in Autism

**DOI:** 10.3389/fncel.2015.00519

**Published:** 2016-01-19

**Authors:** Janet K. Kern, David A. Geier, Lisa K. Sykes, Mark R. Geier

**Affiliations:** ^1^Institute of Chronic Illnesses, Inc., Silver SpringMD, USA; ^2^CoMeD, Inc., Silver SpringMD, USA

**Keywords:** neuroinflammation, encephalitis, autism spectrum disorder, microglia, astrocytic activation, cytokines, regression

## Abstract

In recent years, many studies indicate that children with an autism spectrum disorder (ASD) diagnosis have brain pathology suggestive of ongoing neuroinflammation or encephalitis in different regions of their brains. Evidence of neuroinflammation or encephalitis in ASD includes: microglial and astrocytic activation, a unique and elevated proinflammatory profile of cytokines, and aberrant expression of nuclear factor kappa-light-chain-enhancer of activated B cells. A conservative estimate based on the research suggests that at least 69% of individuals with an ASD diagnosis have microglial activation or neuroinflammation. Encephalitis, which is defined as inflammation of the brain, is medical diagnosis code G04.90 in the International Classification of Disease, 10th revision; however, children with an ASD diagnosis are not generally assessed for a possible medical diagnosis of encephalitis. This is unfortunate because if a child with ASD has neuroinflammation, then treating the underlying brain inflammation could lead to improved outcomes. The purpose of this review of the literature is to examine the evidence of neuroinflammation/encephalitis in those with an ASD diagnosis and to address how a medical diagnosis of encephalitis, when appropriate, could benefit these children by driving more immediate and targeted treatments.

## Introduction

Autism or autism spectrum disorder (ASD) is a childhood neurodevelopmental disorder that is behaviorally defined and psychiatrically diagnosed based on a spectrum of qualitative impairments in social interaction and communication, and in restricted and stereotyped patterns of behavior, interests, and activities (American Psychiatric Association, 2013). In addition, children diagnosed with an ASD diagnosis have a high prevalence of various co-morbid medical conditions (Banaschewski et al., 2011; Geier et al., 2012; Ozsivadjian et al., 2014). Despite this fact, an ASD diagnosis still remains under the diagnostic criteria of a purely psychiatric disorder.

In recent years, many studies indicate that children with an ASD diagnosis have brain pathology suggestive of ongoing neuroinflammation or encephalitis (encephalitis is defined as brain inflammation) in different regions of the brain (Enstrom et al., 2005; Pardo et al., 2005; Vargas et al., 2005; Zimmerman et al., 2005; Chez et al., 2007; Morgan et al., 2010, 2012; Tetreault et al., 2012). Encephalitis is medical diagnosis code G04.90 in the International Classification of Disease, 10th revision, clinical modification (ICD-10-CM). However, even though the research indicates that the brain inflammation is relatively common in these children, children with an ASD diagnosis are not generally given the medical diagnosis of encephalitis. Instead, they continue to be diagnosed using purely psychiatric diagnostic codes. This may be due, in part, to original misconceptions about the disorder when it was first identified in 1943 by Leo Kanner, who attributed it to the emotional unavailability of the affected child’s mother. While erroneous, this misconception originally and lastingly branded autism as a psychiatric disorder. Now, despite decades of published scientific and medical literature documenting its physical symptoms, autism is most often still treated as a psychiatric condition with psychiatric medications.

As a result, critical new research into the physical symptoms of autism is often neglected. Additionally, the time lag between new research findings being published and their being incorporated into medical practice standards further contributes to a delay in the recognition and treatment of the physical symptoms of autism or ASD. It is crucial that new landmark findings from neuroscience, about the brain pathology found in ASD, be translated into the practice of medicine because this will drive new medically directed and targeted treatments, which may be more effective and safer than previous interventions guided by psychiatric labels. The current use and safety of psychiatric medications in autism and ASD will be discussed later in this paper.

The purpose of this review of the literature, is to examine the evidence of neuroinflammation/encephalitis in those with an ASD diagnosis and to address how a medical diagnosis of encephalitis could benefit these children by driving more immediate and targeted treatments. The review begins with evidence of neuroinflammation in ASD. Although there are numerous studies that show markers of systemic inflammation in ASD (Rossignol and Frye, 2014), this review focuses on the brain and cerebral spinal fluid (CSF).

## Autism, Neuroinflammation, and Encephalitis

As mentioned previously, there are many studies showing children with an ASD diagnosis have ongoing neuroinflammation/encephalitis in different regions of the brain (see **Table 1**; Enstrom et al., 2005; Pardo et al., 2005; Vargas et al., 2005; Zimmerman et al., 2005; Chez et al., 2007; Morgan et al., 2010, 2012). Active neuroinflammatory processes are found throughout the brain in both the cerebral cortex and in the cerebellum of patients with autism (Vargas et al., 2005). Of critical importance to this issue is that post-mortem brain tissue studies examining the brains of children with an ASD diagnosis reveal significant evidence of neuroinflammation/encephalitis regardless of the child’s age, indicating that they were suffering from sustained neuroinflammation/encephalitis processes. As Herbert (2005) described, brain abnormalities in those diagnosed with an ASD reveal significant ongoing neuroinflammation to be a central element of the observed brain pathology.

**Table 1:** Evidence of neuroinflammation/encephalitis in the brains and cerebral spinal fluid (CSF) of subjects with autism spectrum disorder (ASD).

Studies	N Case/Control	Findings	Researchers’ Conclusion
Vargas et al., 2005	15/12	(1) Marked activation of microglia and astroglia, and cytokine profiling indicated that macrophage chemoattractant protein (MCP)-1 and tumor growth factor-beta1, derived from neuroglia, were the most prevalent cytokines in brain tissues	Active neuroinflammatory process in those with an ASD diagnosis
	6/9	(2) CSF showed a unique proinflammatory profile of cytokines, including a marked increase in MCP-1	
Li et al., 2009	8/8	Proinflammatory cytokines (TNF-alpha, IL-6, and GM-CSF), Th1 cytokine (IFN-gamma) and chemokine (IL-8) were significantly increased in the brains of ASD patients compared with the controls	Brain inflammation in those with an ASD diagnosis and autoimmune disorder
Young et al., 2011	9/9	Neurons, astrocytes, and microglia all demonstrated increased extranuclear and nuclear translocated NF-κB p65 expression in brain tissue from ASD donors relative to samples from matched controls	Part of a putative molecular cascade leading to brain inflammation
Morgan et al., 2010	13/9	Microglial activation and increased microglial density in the dorsolateral prefrontal cortex in those with autism	Neuropathological alteration and brain inflammation
Morgan et al., 2012	13/9	Microglia are more frequently present near neurons in the autism cases at a distance interval of 25 μm, as well as 75 and 100 μm	Aberrantly close microglia-neuron association in the ASD disorder
Wei et al., 2011	6/6	Interleukin (IL)-6 increased in the cerebellum of autistic subjects	Localized inflammation of the central nervous system
Tetreault et al., 2012	11/12	Individuals with autism had significantly more microglia compared to controls in the fronto-insular and visual cortex	The brain’s immune cells (microglia) are probably denser throughout cerebral cortex in ASD
Suzuki et al., 2013	20/20	Excessive microglial activation in multiple brain regions in young adult subjects with an ASD diagnosis was found using regional brain [11C](R)-PK11195 binding potential as a representative measure of microglial activation	Augmented but not altered microglial activation (brain immune-cell activation), which is indicative of pro-inflammatory processes in the brain
Fatemi et al., 2008	24/22	The levels of recognized indicators of inflammatory processes in brain tissue, including Aquaporin 4 and Connexin 43 were examined in the brains of those with an autism diagnosis. The study found that, in contract to controls, in evaluations using the brain’s β-actin level as a reference, Aquaporin 4 expression was decreased significantly in cerebellum, while, in Brodmann’s area 9 (superior frontal cortex), Connexin 43 was elevated in the brains of those diagnosed with autism.	Inflammatory processes in ASD
Chez et al., 2007	10	Elevation of cerebrospinal fluid levels of TNF-α was significantly higher (mean = 104.10 pg/mL) than concurrent serum levels (mean = 2.78 pg/mL)	Indicative of CNS inflammatory mechanisms
Laurence and Fatemi, 2005	3	Elevated levels of GFAP in the frontal, parietal, and cerebellar cortices using age-matched autism and control post-mortem brain specimens	Indicative of microglial and astroglial activation


			Increased GFAP levels signify gliosis, reactive injury in those with an ASD diagnosis
Rosengren et al., 1992	47/13	GFAP levels in CSF in children with autism were higher than those in normal control children	Indicate reactive astrogliosis in the CNS
Ahlsen et al., 1993	47/25	Average levels of GFAP in the CSF of children with autism three times higher than control group	Reactive gliosis
Bailey et al., 1998	6/8	Cerebellum in autism showed an increase in GFAP	Reactive gliosis
López-Hurtado and Prieto, 2008	8/7	The mean density of glial cells was greater in the autistic cohort than controls in area 22 (*p* < 0.001), area 39 (*p* < 0.01), and area 44 (*p* < 0.05)	Results are consistent with accelerated neuronal death in association with gliosis and lipofuscin accumulation
Rose et al., 2012	12/12	3-chlorotyrosine (3-CT; an established biomarker of a chronic inflammatory response) significantly increased in autism cerebellum and BA22	Chronic inflammatory response
Crawford et al., 2015	14/14	Levels of GFAP immunoreactivity were significantly elevated (P = 0.008) in anterior cingulate cortex (Brodmann area 24; BA24) white matter of ASD compared to controls	Activation of white matter astrocytes in the anterior cingulate cortex as a result of a yet undefined cellular insult

These findings, showing evidence of inflammation in brain tissue in ASD, are evidenced by biomarkers of inflammation/encephalitis in the CSF and blood of individuals diagnosed with an ASD (Zimmerman et al., 2005; Chez et al., 2007). Neuroinflammation, in general, is characterized by the reactivity of microglial cells and astrocytes, activation of inducible NO-synthase (i-NOS), and increased expression and/or release of cytokines and chemokines (Monnet-Tschudi et al., 2011). All of these neuroinflammatory processes have been observed in those with an ASD diagnosis. **Table 1** summarizes evidence supporting the presence of neuroinflammation/encephalitis in the brains and CSF of select individuals who have an ASD diagnosis. To date, there are at least 16 studies which reveal neuroinflammation to be an element of the ASD pathology. The following section discusses the specific biomarkers of neuroinflammation found in ASD, and their relevance and interplay.

### Biomarkers of Neuroinflammation in ASD

In those with an ASD diagnosis, some important biomarkers indicative of brain inflammation, include (but are not limited to): (1) microglial and astrocytic activation (Vargas et al., 2005); (2) a proinflammatory profile of cytokines (Vargas et al., 2005); and (3) nuclear factor kappa-light-chain-enhancer of activated B cells (NF-κB) activation (Young et al., 2011). The presence of one or more of these biomarkers can influence and potentiate the others. Moreover, activated microglial and astrocytes, proinflammatory cytokines, and aberrant NF-κB activity can ultimately create an environment of excessive brain inflammation, which can lead to destruction of critical brain tissue (Rodriguez and Kern, 2011). In other words, those conditions can intensify brain inflammation making matters worse. A brief explanation is as follows.

#### Microglia

Microglia are a type of glial cell. They are the resident macrophages in the central nervous system (CNS) and act as the first and main form of active immune defense in the brain and spinal cord. Microglia as an innate immune response cell react in a proinflammatory fashion to attack infectious agents or altered proteins/cells, but then shift to a more anti-inflammatory phenotype to remove debris and repair the damage. Microglia can play both a beneficial and a detrimental role, and thus it is not easy to separate their contributions in disease onset and progression (Carson et al., 2007).

Vargas et al. (2005) and several others (e.g., Pardo et al., 2005; see **Table 1**) reported that individuals who had an ASD diagnosis had neuroinflammatory processes present in both brain tissue and/or CSF and that microglial activation appeared to be part of a sustained neuroinflammatory process. Unfortunately however, when the neuroinflammatory process is sustained, microglial activation can contribute to disease progression which can result in loss of healthy brain tissue (Rogers et al., 2007; Smith et al., 2012). In a sustained neuroinflammatory state, microglia can adopt an amoebic phenotype and start engulfing synapses and other healthy brain tissue (Rodriguez and Kern, 2011). The consequence of synapses and other neuronal tissue being engulfed is cell loss and reduced connectivity, both of which are found in the brains of those with an ASD diagnosis (Rodriguez and Kern, 2011).

#### Astroglia or Astrocytes

Although the inflammatory responses in the CNS are primarily mediated by microglia, evidence suggests that astrocytes (also a type of glial cell in found in the brain) are key regulators of neuroinflammation in the CNS (Guerra et al., 2011; Cekanaviciute et al., 2014). Astrocytes are found to be activated in those with an ASD diagnosis. Moreover, when astrocytes are hypertrophic and proliferative, they up-regulate the expression of glial fibrillary acidic protein (GFAP; Stichel and Muller, 1998), and GFAP is also found to be elevated in the brains and CSF in those with an ASD diagnosis (Ahlsen et al., 1993; Laurence and Fatemi, 2005; see **Table 1**). Ahlsen et al. (1993), for example, observed that GFAP levels were threefold higher in the CSF of children diagnosed with an ASD in comparison to controls.

#### Cytokines

Cytokines are small proteins that are used in cell signaling, and they include: chemokines, interferons, interleukins, lymphokines, and tumor necrosis factor (TNF). Proinflammatory cytokines promote inflammation. In those diagnosed with an ASD, the brain and CSF have a unique and elevated proinflammatory profile of cytokines in comparison to controls (Vargas et al., 2005; Chez et al., 2007; Li et al., 2009; see **Table 1**). As reported by Li et al. (2009), proinflammatory cytokines [TNF-alpha, interleukin (IL)-6 and granulocyte-macrophage colony-stimulating factor], Th1 cytokine (interferon-gamma) and chemokine (IL-8) are increased in the brains of individuals with ASD. Proinflammatory cytokines are also found to be increased in the peripheral blood of those with an ASD diagnosis in comparison to controls (Zimmerman et al., 2005; Molloy et al., 2006).

#### Nuclear Factor Kappa-Light-Chain-Enhancer of Activated B Cells (NF-κB)

NF-κB is a protein found in almost all cell types. This protein is a transcription factor that will promote gene expression of several inflammatory mediators. It mediates the regulation of cellular immune responses by promoting the expression of inflammatory cytokines and chemokines and by establishing a feedback mechanism that can produce chronic or excessive inflammation (Young et al., 2011). Thus, when NF-κB becomes aberrantly active, it has the potential to produce chronic or excessive inflammation (Young et al., 2012). NF-κB activation induces numerous proinflammatory gene products including cytokines, cyclooxygenase-2 (COX-2), and inducible nitric oxide synthase (iNOS; Park and Youn, 2013). In individuals with an ASD diagnosis, Young et al. (2011) found NF-κB is aberrantly expressed in the orbitofrontal cortex, in comparison to controls, as part of a molecular cascade leading to inflammation, especially of resident immune cells in brain regions that are associated with the behavioral and clinical symptoms of those with an ASD diagnosis. Although, one study did not find aberrant NF-κB in the brains of children with ASD (Malik et al., 2011), peripheral blood markers confirm abnormal NF-κB activity. Naik et al. (2011) for example, evaluated for NF-κB in peripheral blood samples of 67 children with autism and 29 control children and found a significant increase in NF-κB DNA binding activity in the peripheral blood samples of children with autism. They further stated that autism may arise, at least in part, from an NF-κB pathway gone awry. Other studies corroborate their findings (Ziats and Rennert, 2011).

The research findings in this section suggest that excessive neuroinflammation is an element of the neuropathology in those with an ASD diagnosis. The following section estimates the percentage of children with an ASD diagnosis who may be affected.

### Estimation of the Percentage of Children with an ASD Diagnosis Who are Affected

The percentage of children with an ASD diagnosis who have neuroinflammation/encephalitis remains unclear. Due to the state of various subtypes in ASD, some children diagnosed with an ASD may not have neuroinflammation. However, evidence from clinical research suggests that neuroinflammation is common among those with an ASD diagnosis. An examination of the evidence supporting a link between neuroinflammation and autism that might be used to estimate the percentage of children affected follows.

Of the studies that examined neuroinflammatory biomarkers in the brain and CSF of those with an ASD diagnosis, most suggested that all of the children examined showed signs of brain inflammation (Vargas et al., 2005). In some studies, the authors overtly concluded that the neuroinflammation was found in all of the individuals they examined (Chez et al., 2007; López-Hurtado and Prieto, 2008; Rose et al., 2012). For example, Rose et al. (2012), who studied brain samples from 12 children with autism and 12 controls, mentioned that all of the markers examined were significantly altered in autism, including 3-chlorotyrosine (3-CT), an established biomarker of a chronic inflammatory response, which was significantly increased. López-Hurtado and Prieto (2008) also mentioned, in their study of eight individuals with autism and seven controls, that the autistic subjects of all ages demonstrated greater density of glial cells in comparison to controls (up to double).

However, there are a few studies where the authors mentioned that the biomarkers of neuroinflammation studied were found in some but not all of the individuals examined. For example, Morgan et al. (2010) examined the dorsolateral prefrontal cortex of male cases diagnosed with autism (*n* = 13). The authors stated that the microglia were activated in 9 of 13 cases with autism (69%). Tetreault et al. (2012) observed all but one individual diagnosed with an ASD (out of the 11 studied) had higher levels of microglial activation than controls. Thus, 91% showed microglial activation or neuroinflammation. However, Tetreault et al. (2012) also stated that the one individual without the microglia activation or neuroinflammation was an outlier, behaviorally, with respect to other individuals diagnosed with autism and examined.

Thus, based on the available research, a conservative estimate suggests that at least 69% of individuals with an ASD diagnosis have microglial activation or neuroinflammation. However, given the lower number of subjects analyzed in each of the presented studies, this estimate should be considered with care. The actual percentage could conceivably be more or less. For a more accurate estimate, a larger study is needed – one that quantitatively examines multiple regions of the brain for glial activation in concert with an assessment of other markers of activation (e.g., cytokines); this would permit researchers to determine more precisely the frequency/percentage of individuals with an ASD diagnosis who also show microglial activation.

## How Neuroinflammation May Contribute to the Development Of ASD: Regression, Encephalitis, and Clinical Symptoms

Knowledge of the effects of sustained and exaggerated neuroinflammation and microglia activation on brain connectivity is critical to understand how neuroinflammation could contribute to the development of an ASD. Sustained and exaggerated microglial activation can lead to cell loss and loss of connectivity. As mentioned earlier, in a sustained neuroinflammatory state, microglia can adopt an amoebic phenotype and start engulfing synapses and other healthy brain tissue with deleterious consequences for neurons and synaptic architecture (Lu et al., 2011; Rodriguez and Kern, 2011). Furthermore, when microglia are triggered to switch to an inflammatory phenotype, not only can this lead to microgliosis and neuroinflammation resulting in a disruption of normal neuroimmune homeostasis, but also this detrimental process can continue long after the initial insult or cause for the activation has been resolved (Lu et al., 2011).

As mentioned, the consequence of sustained microglial activation is cell loss and reduced connectivity, both of which are found in the brains of those with an ASD diagnosis (Rodriguez and Kern, 2011). An examination of the scientific literature in ASD clearly shows that connectivity is disrupted (Wass, 2011). Numerous studies show loss of connectivity in ASD (Kern et al., 2015). In addition, the issues of connectivity in ASD have been shown to correlate with ASD symptom severity – the greater the cell loss and connectivity issues, the worse the ASD symptom severity (Kikuchi et al., 2014; Kern et al., 2015). Neuronal cell loss and reduced connectivity could understandably lead to neurological loss of skills and abilities or regression. Once a threshold of sufficient neuronal cell loss and neuronal disconnection has been reached, a child would then become clinically symptomatic, i.e., show signs of regression or loss of skills and abilities.

In addition, astroglial activation, usually associated with chronic neuroinflammation and found in ASD, has beneficial as well as detrimental effects (Kern et al., 2012; Skripuletz et al., 2013). Astrogliosis is sometimes accompanied by microgliosis and demyelination (Skripuletz et al., 2013). Neuronal demyelination could also lead to neurological loss of skills and abilities and possibly characterize the regression scenario in ASD.

The concept of regression (loss of previously acquired skills and abilities) in some children with ASD has been validated by many studies (Tuchman, 1996; Davidovitch et al., 2000; Goldberg et al., 2003; Ozonoff et al., 2005, 2010; Werner and Dawson, 2005; Hansen et al., 2008; Stefanatos, 2008; Malhi and Singhi, 2012; Kern et al., 2014a,b). For example, Werner and Dawson (2005) evaluated home videotapes of children with autism between their first and second birthday parties with and without a reported history of regression, as well as videotapes of typically developing children. Analyses revealed that infants diagnosed with an ASD with regression show similar use of joint attention and more frequent use of words and babble compared with typical infants at 12 months of age. In contrast, infants diagnosed with an ASD characterized by early onset of symptoms and no regression displayed fewer joint attention and communicative behaviors at 12 months of age. By 24 months of age, both groups of toddlers diagnosed with an ASD displayed fewer instances of word use, vocalizations, declarative pointing, social gaze, and orienting to name as compared with typically developing 24-month-olds.

According to Ozonoff et al. (2005), children with ASD can be divided into three groups: an early onset group, a definite regression group, and a heterogeneous mixed group (delays-plus-regression). They, for example, found that approximately 52% had regressed. Similarly, in a study of 135 children with ASD, Kern et al. (2014a) also found that children with ASD could be divided into these three groups of children and reported that 61% were reported to have regressed. The skills most frequently reported to be lost were speech, eye contact, pointing, and socialization. Other skills mentioned were non-verbal communication, responsiveness, interest in others, expression, ability to imitate and, to a much lesser extent, motor skills. Problems noted were tantrums, behavioral issues, apparent deafness, and sensory issues (oversensitivity and undersensitivity).

## Considering the Possible Forms and Causes of Encephalitis in ASD

According to the Encephalitis Society, encephalitis is inflammation of the brain, and this inflammation is caused by either an infection invading the brain (Infectious Encephalitis) or the immune system attacking the brain in error (post-infectious or Autoimmune Encephalitis; Encephalitis Society, 2015). Autoimmune Encephalitis usually follows a viral infection (such as those that cause rashes in childhood) or immunizations. However, it has been recognized recently that there are other types of Autoimmune Encephalitis resulting from the brain being attacked by the body’s immune system. Some of these types of Autoimmune Encephalitis include Potassium channel complex antibody associated Encephalitis, *N*-methyl D-aspartate (NMDA) receptor Encephalitis, and Hashimoto’s Encephalitis.

Importantly, in cases of brain inflammation from infection, the pathogen does not necessarily have to enter the brain. Brain inflammation (and microglia) can be activated by systemic infection and inflammation (Teeling and Perry, 2009). For example, inflammatory stimuli in the periphery [e.g., lipopolysaccharide (LPS) and inflammatory cytokines] can induce transcripts for interleukin (IL)-1b, IL-6, and tumor necrosis factor-a (TNF-a) in discrete brain areas (Ban et al., 1992; Laye et al., 1994). Thus, pathogens and even peripheral cytokines need not enter the brain to elicit changes. As described by Jang and Johnson (2010), cells associated with the peripheral innate immune system (e.g., macrophages and monocytes) can produce inflammatory cytokines such as IL-1b, IL-6, and TNF-a that facilitate communication between the periphery and the brain during infection. Additionally, several studies find that peripheral cytokines can enter the brain (Banks and Kastin, 1991; Gutierrez et al., 1993; Banks et al., 1994a,b, 1995).

Systemically produced pro-inflammatory mediators can signal the brain, leading to activation of microglial cells; and even though this process can be a normal part of our defense (and in most individuals causes no damage to neurons), in some susceptible individuals the systemic inflammation leads to inflammatory responses in the brain and increased neuronal death (Teeling and Perry, 2009). Evidence indicates that this may be the case in some cases of ASD. As mentioned in a previous section, sustained brain inflammation is found in ASD, as well as neuronal cell loss (Kern et al., 2013). In addition, studies show that children with ASD have elevated blood inflammatory markers. For example, Masi et al. (2015) completed a meta-analysis on studies comparing plasma and serum concentrations of cytokines in unmedicated individuals with ASD and controls, and they found significantly altered concentrations of cytokines in ASD. They stated that the findings strengthen the evidence of an abnormal cytokine profile in ASD where inflammatory signals dominate.

Based on this information, it is possible that an element of encephalitis or neuroinflammation exists in ASD and can be characterized as Post Infectious Encephalitis secondary to a systemic infection. Notably, regression in ASD is sometimes reported to follow fever, rashes, infection, and immunizations (Kern et al., 2014b). However, there is also evidence for Autoimmune Encephalitis such as NMDA Encephalitis, and there are documented cases of NMDA Encephalitis in ASD (which will be discussed in more detail later).

Having an autoimmune type encephalitis in ASD is certainly biologically plausible. Children with ASD have an elevated prevalence of specific immune-related comorbidities, such as allergies and autoimmune diseases (Zerbo et al., 2015). Symptoms of immune dysfunction in ASD include (but are not limited to): neuroinflammation, presence of autoantibodies, increased T cell responses, and enhanced innate NK cell and monocyte immune responses, etc. (Mead and Ashwood, 2015). Moreover, these responses are frequently associated with more impairment in core ASD features such as impaired socialization and communication, and repetitive and abnormal behaviors (Mead and Ashwood, 2015).

Interestingly, neurotoxic effects and neuroinflammation were observed in young Wistar rats that were injected (intracerebroventricularly) with autism sera within hours after birth. According to Kazim et al. (2015), the rats injected with the autism sera demonstrated developmental delay and deficits in social communication, interaction, and novelty. The neurobiological changes and the behavioral autistic features were ameliorated by treatment with a ciliary neurotrophic factor (CNTF) small peptide mimetic, Peptide 6 (P6), which is known to have neuroprotective effects (Kazim et al., 2015).

From the evidence presented in this section, it may be plausible that encephalitis in ASD has various underlying factors. Importantly, studies which report a regression of patients into an ASD diagnosis following encephalitis include infectious encephalitis, post-infectious or Autoimmune Encephalitis, or purely Autoimmune Encephalitis (such as NMDA Encephalitis; Ghaziuddin et al., 2002; Creten et al., 2012; González-Toro et al., 2013; Marques et al., 2014; Scott et al., 2014). What the possible external triggers of this inflammatory process in children with autism may be is the subject of the following section.

## Possible External Triggers of the Inflammation Process in Autism

As mentioned earlier, systemic inflammation and/or infection can gain access to the CNS via blood flow and elicit an inflammatory response the brain (Sankowski et al., 2015). The resulting inflammatory mediators could interfere with neuronal and glial well-being, leading to a disruption in brain homeostasis and persistent inflammation and immune activation in the brain, ultimately resulting in cognitive and behavioral manifestations (Sankowski et al., 2015). Thus, it is plausible that systemic inflammation and/or infection could trigger the inflammation or encephalitis seen in the brains of children with an ASD.

In addition, the production of brain autoantibodies, notably found in children with ASD and in specific cases of autistic regression and encephalitis, could also be secondary to various types of external triggers. Researchers have suggested various exposures that can contribute to the production of brain autoantibodies in autism (Mostafa and Refai, 2007; Mostafa and Al-Ayadhi, 2015).

To this point, a study by Vojdani et al. (2003) provides evidence to support the hypothesis that there are external triggers, such as the ones mentioned previously, that can instigate the production of brain autoantibodies in children with autism. Vojdani et al. (2003) measured IgG, IgM, and IgA antibodies against CD26, CD69, streptokinase (SK), gliadin, and casein peptides and against ethyl mercury bound to human serum albumin in patients with autism. From the results, they proposed that bacterial antigens (SK), dietary peptides (gliadin, casein) and Thimerosal (ethyl mercury) in individuals with pre-disposing HLA molecules, bind to CD26 or CD69 and induce antibodies against these molecules. Their study demonstrated that dietary peptides, bacterial toxins, and xenobiotics bind to lymphocyte receptors and/or tissue enzymes, resulting in and autoimmune reaction in children with autism.

It is conceivable that this process could begin with a single exposure or trigger; however, a combination of exposures or factors could also trigger a cascade of events resulting in brain inflammation and production of brain autoantibodies. Numerous studies have shown that toxins and pathogens can work synergistically – where the effect of the combination of their presence is greater than the sum of their individual effects (Kern et al., 2012).

## How Recognizing Encephalitis or Brain Inflammation as a Potential Component of ASD May Improve Treatment

### Issues with Current Mainstream Treatments and Therapies

As previously mentioned, another benefit of recognizing encephalitis in those with an ASD diagnosis is that this recognition might lead to more targeted and potentially more effective medical treatments. The current mainstream treatments and therapies for those with an ASD diagnosis emphasize educational interventions such as applied behavioral analysis (ABA) and/or psychoactive drugs such as Risperdal. These types of treatments do not address the patient’s underlying medical illness or disease pathology. This may be the reason for the dismal findings reported in a 2011 article in Pediatrics by Al-Qabandi et al. (2011). These researchers reported that although there are many available therapeutic approaches to childhood autism, none are curative or have well-established efficacy. This finding continues even with the promotion of early intervention.

In addition, the psychiatric or antipsychotic medications frequently prescribed in ASD (e.g., risperidone or Risperdal), have serious side effects. For example, antipsychotics such as Risperdal can cause: neuroleptic malignant syndrome (a potentially fatal reaction); tardive dyskinesia (abnormal facial, shoulder and limb movements, which can be permanent); breast swelling or tenderness; low white blood cells; low platelets; high blood sugar; and many other serious side effects (PDR.net, 2014).

### Treatment Response and Timeliness

Recent research suggests that even though parental concerns that their child might have ASD are generally expressed early on and reliably (Kern et al., 2014a,b; Sacrey et al., 2015), common responses to these concerns from healthcare providers are often reassuring or passive which subsequently delays diagnosis and treatment (Zuckerman et al., 2015). Another possible benefit of recognizing encephalitis or brain inflammation as a potential component of ASD is that this may drive a more timely response. If providers understand that the affected child may have an identifiable medical condition which would respond to appropriate and prompt medical treatment, they may be more likely to initiate medical intervention.

### Evidence to Suggest Effectiveness in Treating Encephalitis in ASD

Several studies that link encephalitis with the onset of autism or an ASD, also report the improvement or amelioration of autism/ASD symptoms when the encephalitis was treated (González-Toro et al., 2013; Scott et al., 2014). For example, Scott et al. (2014) reported on a 33-month-old boy who presented with irritability, insomnia, decreased appetite, and symptoms of autistic regression following an upper respiratory tract infection. He displayed loss of previously acquired skills, including: language (eventually becoming mute and non-communicative), the ability to interact socially, and eye contact. The child was found to have anti-NMDA receptor encephalitis. Treatment with intravenous (IV) immunoglobulins and steroids resulted in the child’s reacquisition of language and social skills and in the resolution of his abnormal movements. According to the authors, reacquisition of language and social skills were observed after the third day of treatment. He also began to show interest in his parents again and his eye contact improved. After the initial IV treatment was completed, he was started on high-dose steroids (2 mg/kg/d) for 2 weeks, with a slow tapering off over the next 6 weeks. During that time, he continued to make significant improvements and his behavior and personality were restored to their pre-illness state. In addition, he regained the ability to use multiple short phrases.

A similar case (González-Toro et al., 2013) involved a 5 years old female who lost previously acquired skills and evolved into autism. After showing positive anti-NMDA receptor antibodies in her cerebrospinal fluid, she was diagnosed with anti-NMDA receptor encephalitis. An intravenous perfusion of corticoids, immunoglobulins, and rituximab was used. According the researchers, the child essentially recovered except for a slight language disorder that was still noted 6 months after treatment.

Thus, as these reports suggest, another benefit to recognizing encephalitis as a physical condition in those with an ASD diagnosis is that it might lead to more targeted and possibly more effective medical treatments. As suggested by McDougle and Carlezon (2013), addressing the neuroimmunological pathophysiology in ASD offers exciting new possibilities for therapy.

In the aforementioned cases, the drugs used were intravenous steroids and immunoglobulins (and rituximab). However, there are several studies which report on other pharmaceutical and nutraceutical treatments that can reduce microglial activation and/or the levels of their associated inflammatory cytokines. If encephalitis were routinely assessed as a component in autism and ASD, then in those cases where it is identified, more benign treatments might be available than the current psychiatric medication choices, and their use might be more efficacious in producing a positive outcome. The authors of this review are not recommending any treatment in particular, and acknowledge that, in any treatment regimen, the risk/benefit ratio would have to be considered. That said, further research into the diagnosis and treatment of encephalitis as a potential component of ASD is merited.

## Discussion

The dramatic rise in ASD began in the 1990s, and in the past two decades, the rates of ASD have increased by 289% (Boyle et al., 2011). The sudden and dramatic rise in ASD prevalence has, in some ways, caught the medical community “off guard.” In the midst of the meteoric rise in rates of autism and ASD, significant new research into the physical symptoms has been done. The challenge now is to incorporate this new research about the physical symptoms of autism into the practice of medicine that historically has stereotyped autism as a purely psychiatric disorder. For the benefit of patients, the physical symptoms of autism must be recognized and treated. For children with ASD, particularly those who have begun to regress into ASD and show other signs of neurological regression, testing for encephalitis may be warranted. Particularly, given the documented cases of children with regressive ASD and NMDA Encephalitis who tested positive for anti-NMDA receptor antibodies, routine testing for anti-NMDA receptor antibodies in ASD should be seriously considered. The study by Scott et al. (2014), mentioned earlier, of the child who regressed into autism and recovered from treatment for NMDA, indicates that there is benefit to recognizing the possibility of encephalitis in children with ASD. The delay in incorporating new research findings into medical practice standards is unfortunate because if a diagnosis of autism or ASD were recognized in the medical community as having a possible component of encephalitis that could be tested and treated appropriately, such treatment for encephalitis would likely reduce, and possibly eliminate, ASD symptoms in some children. Future studies should include treatments for neuroinflammation in ASD.

## Author Contributions

JK was the main writer and analyzed data, DG reviewed the manuscript structure, ideas and science, LS co-wrote and edited structure, ideas, and science, and MG evaluated and reviewed manuscript ideas and science. All authors read and approved the final manuscript.

## Conflict of Interest Statement

The authors have been involved in vaccine/biologic litigation. The authors declare that the research was conducted in the absence of any commercial or financial relationships that could be construed as a potential conflict of interest.
